# A preliminary study of the association between *Blastocystis* and quantification of selected yeasts in IBD and IBS patients

**DOI:** 10.3389/fmed.2025.1514587

**Published:** 2025-02-13

**Authors:** Zohre Khosravany, Sadegh Khodavaisy, Alireza Olyaiee, Amir Sadeghi, Sara Nemati, Shabnam Shahrokh, Sara Mohammad Ali Gol, Sajad Shojaei, Hanieh Mohammad Rahimi, Hamed Mirjalali

**Affiliations:** ^1^Foodborne and Waterborne Diseases Research Center, Research Institute for Gastroenterology and Liver Diseases, Shahid Beheshti University of Medical Sciences, Tehran, Iran; ^2^Department of Medical Parasitology & Mycology, School of Public Health, Tehran University of Medical Sciences, Tehran, Iran; ^3^Department of Biology, Science and Research Branch, Islamic Azad University, Tehran, Iran; ^4^Gastroenterology and Liver Diseases Research Center, Research Institute for Gastroenterology and Liver Diseases, Shahid Beheshti University of Medical Sciences, Tehran, Iran; ^5^Basic and Molecular Epidemiology of Gastrointestinal Disorder Research Center, Research Institute for Gastroenterology and Liver Diseases, Shahid Beheshti University of Medical Sciences, Tehran, Iran

**Keywords:** irritable bowel syndrome, inflammatory bowel diseases, calprotectin, mycobiome, *Blastocystis*, *Candida albicans*, *Saccharomyces cerevisiae*

## Abstract

**Objective:**

Irritable bowel syndrome (IBS) and inflammatory bowel disease (IBD) are gastrointestinal disorders, which can be triggered by gut microbiota dysbiosis. The development of IBS-like symptoms has been linked to the overgrowth of *Candida* spp. In addition, the critical role of fungi has been highlighted in the pathogenesis of IBD. This study investigated the association between *Blastocystis* and selected yeasts in IBS and IBD patients.

**Methods:**

This investigation is a cross-sectional study from 2022 to 2024, performed on 91 participants, including 20 healthy individuals, 27 patients with IBS, and 44 IBD patients [39 with ulcerative colitis (UC; 88.63%) and 5 (11.37%) Crohn’s disease (CD)], who were also categorized based on the presence of *Blastocystis*. Total DNA was extracted from stool samples, and the presence and quantity of yeasts including *C. albicans, C. tropicalis, C. glabrata, C. parapsilosis, C. krusei, Geotrichum candidum, Rhodotorula* spp., *Cryptococcus neoformans*, and *Saccharomyces cerevisiae* were evaluated by real-time PCR. Statistical tests were used to assess significant associations between variables.

**Results:**

*Saccharomyces cerevisiae* and *C. albicans* were the most prevalent yeasts in all groups. *Candida tropicalis* and *C. neoformans* were identified in neither patients nor healthy subjects. The presence/absence of *C. albicans* was not significantly different between patients with IBD, IBS, and the control groups. This was similar for *G. candidum*. However, there was a difference in the presence of *S. cerevisiae* among patients, although it was insignificant (*p*-value = 0.077). There was a significant difference in the quantity of *C. albicans* between IBD (880.421 ± 2140.504), IBS (10.307 ± 15.206), and controls (2875.888 ± 8383.889) (*p*-value = 0.020). Specifically, the source of difference was seen between IBD patients and the control group (*p*-value = 0.005). In addition, considering the presence of *Blastocystis*, a statistically significant association was seen between the number of *C. albicans* and the sample groups (*p*-value = 0.013). The quantity of *C. albicans* was significantly different between IBS and IBD patients.

**Conclusion:**

Regarding the presence of *Blastocystis*, the quantity of *C. albicans* and *S. cerevisiae* was increased and decreased in the studied groups, respectively. This is a preliminary study, and eukaryote–eukaryote association in IBS and IBD patients should be considered in further studies.

## 1 Introduction

Irritable bowel syndrome (IBS) is one of the most frequently reported conditions among gut–brain interaction (DGBI) disorders (1). The prevalence of IBS varies regarding the diagnostic criteria, from 1.1% for Rome III in Iran to 45% for Rome II in Pakistan (1, 2). According to the latest estimation, the prevalence of IBS in developed countries such as the United States (US) ranges from 4.7 to 5.3% according to the Rome IV criteria (3). In addition, the prevalence of IBS in women seems to be higher than men (2, 4).

The main reason for IBS has not been determined; however, visceral hypersensitivity, gut microbiota alteration, immunological disorders, and increased gut permeability are suggested to be potential agents (5–7). Gut microbiota dysbiosis is thought to trigger visceral hypersensitivity, gut permeability, and immunological disorders in IBS (8, 9). Although metagenomics studies have illustrated that eukaryotes and viruses comprise a small portion of the gut microbiota core, the role of eukaryotes in maintaining the gut’s homeostasis seems to be determinative (10, 11).

Inflammatory bowel disease (IBD) is a well-known gastrointestinal (GI) tract disorder that is described by chronic inflammation. Ulcerative colitis (UC) and Crohn’s disease (CD) are two main conditions in IBD patients, which are characterized based on the regions of involvement through the GI tract and the clinical manifestations (12, 13). Inflammatory bowel disease is thought to be related to Western diets and industrialization. During recent decades, the increased incidence rate of IBD in developing countries infers a drift in the distribution of the disease (14, 15).

Inflammatory bowel disease results from a disturbance in immune responses through the gut. However, the leading hypothesis is an interplay between the gut microbes and the immune system in genetically susceptible patients under special conditions (16). Recent studies have highlighted the crucial role of gut microbiota in the development and amelioration/deterioration of clinical manifestations in patients with IBD (17–19). It was documented that the microbiota significantly contributes to the homeostasis and healthy conditions of the gut (20, 21). Therefore, disturbance in the gut microbiota may provide a proper condition for the development of GI tract disorders such as IBD (21). Fecal calprotectin is a protein, mainly released from neutrophils, which plays a crucial role in regulating innate immunity and responses to intestinal microbes. Measurement of fecal calprotectin indicates mucosal inflammation and inflammatory activity in IBD patients (22).

The role of the gut mycobiome in human health via interaction with bacteria has been recently highlighted (23, 24). It was hypothesized that the composition of the mycobiome and its metabolites may induce dysfunctions in mucosal immunity, leading to gut inflammation (10, 25–27). For example, *β*-glucan, as the major component of the cell wall of fungi, can modulate immune responses and induce inflammation (25). Many studies have evaluated the correlation between fungi and bacteria in healthy and disease conditions (28–30). Compared to the bacteria, in which the cell number per gram of stool may reach 10^11^, the number of fungal cells may vary from 10^5^ to 10^6^ (31, 32). It was demonstrated that a high carbohydrate diet has been weakly associated with overgrowth of yeasts, particularly *Candida* spp. (33), and probably “unexplained clinical symptoms,” which are linked to IBS (11, 34). The promising results of fungicide therapy in mice suffering from visceral hypersensitivity support the critical role of the gut mycobiome in the clinical manifestations of IBS (35). Moreover, metagenomics studies have demonstrated alteration, mainly changes in gut mycobiome species (intra-species or *β*-diversity) in IBS patients compared to healthy controls (23, 36, 37).

Apart from fungi, other eukaryotes, such as parasitic protozoa, seem to be associated with a healthy gut. *Blastocystis* is a hyper-prevalent protist of the human gut, and its prevalence in IBS patients is significantly higher than healthy controls (38, 39). *Blastocystis* is usually reported with lower prevalence in IBD patients compared to healthy subjects, although controversial findings disconfirm these results. *Blastocystis* has been linked to gut microbiota alteration in a broad spectrum of GI disorders (27). Although controversial (40), it was documented that *Blastocystis* is mainly linked to enriched beneficial bacteria of the gut (41, 42). However, there are no data describing intra-kingdom interplay between fungi and parasites in the healthy or unhealthy gut. During recent years, many studies have investigated the bilateral interaction of fungi or *Blastocystis* with the gut bacterial composition in IBS and IBD patients; still, there is no evidence exploring the association between fungi and *Blastocystis* in these patients. The current study aimed to depict a probable association between *Blastocystis* and selected yeasts in IBS and IBD patients.

## 2 Materials and methods

### 2.1 Ethics approval and consent to participate

All experimental protocols followed the ethical principles and the national norms and standards for conducting Medical Research in Iran. The study was performed according to the relevant guidelines and declaration. The current study was also approved by the Research Institute for Gastroenterology and Liver, Shahid Beheshti University of Medical Sciences (SBMU) (IR.SBMU.RIGLD.REC.1403.014 and IR.SBMU.RIGLD.REC.1400.020). Informed consent was also obtained from all participants or their legal guardian(s) before the study.

### 2.2 Sample collection

**Figure 1:**
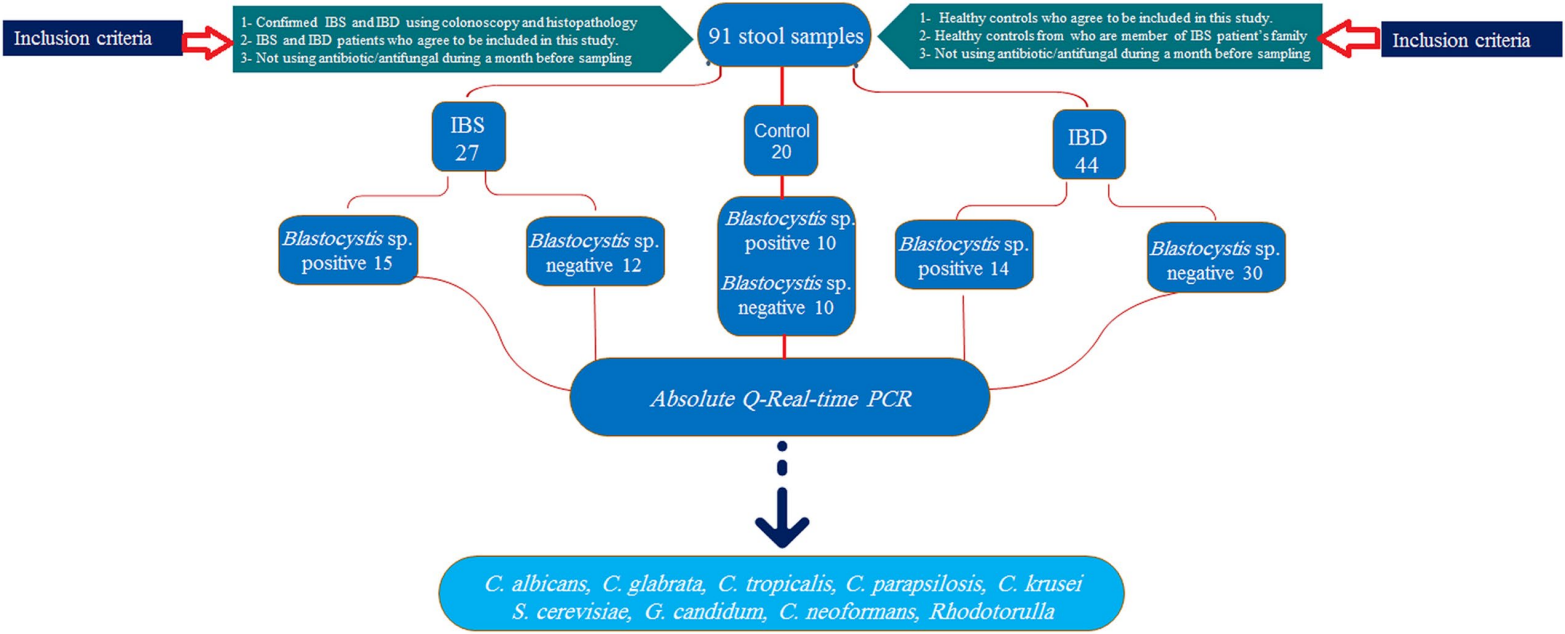
Sampling flowchart of study groups, allocated samples, inclusion criteria, and selected yeasts.

This cross-sectional study was performed on stool samples of 91 subjects consisting of 44, 27, and 20 IBD, IBS, and healthy individuals, respectively, collected from February 2022 to July 2024 (Figure 1). The IBD patients had been confirmed by colonoscopy, and the participants were from patients: (1) newly diagnosed as IBD, (2) patients in remission phase who were referred to the clinic for checkup, and (3) symptomatic patients. Samples were collected from all enrolled patients before consumption of any IBD drugs. Demographic data, drug consumption, stool appearance at the time of sampling, and symptoms during sampling were recorded. Consumption of any antibiotic and systemic antifungal and antiparasitic agents 1 month before sampling was considered exclusion criteria.

Regarding the presence of *Blastocystis*, groups were divided into healthy-*Blastocystis* (10), healthy (10), IBS-*Blastocystis* (15), IBS (12), IBD-*Blastocystis* (14), and IBD (30). Stool samples were immediately transported to the Parasitology lab in the Research Institute for Gastroenterology and Liver Diseases for further analysis. The calprotectin values were considered to distinguish flare and remission in IBD patients. Accordingly, to the clinical consultants, values more than 300 μg/g were considered activity indicators for flare conditions.

### 2.3 Fecal calprotectin

Fecal calprotectin was measured for all IBD samples using an ELISA Calprotectin kit (BÜHLMANN fCAL, Germany) with a working range of 10–3,600 μg/g, as recommended by the instrument. The values of calprotectin were categorized as 1–4, indicating >299, 300–999, 1,000–1999, and >2000 μg/g, respectively.

### 2.4 DNA extraction and *Blastocystis* identification

Total DNA was isolated from all samples using a commercial stool DNA extraction kit (Yekta Tajhiz, Tehran, Iran). In brief, 200 mg of stool samples were washed three times with sterile phosphate buffer saline (PBS; pH = 7.5). After the final washing step, the supernatant was discarded, and the DNA extraction kit treated the pellet. After 30 min incubation of samples at 60°C together with 40 μg of proteinase k and 250 μL of lysis buffer, DNA was isolated and purified according to the instructions. Purified DNA was stored at −20°C until use.

To identify *Blastocystis* in stool samples, the “barcoding region” of the small subunit ribosomal RNA (SSU rRNA) gene was targeted using primers mentioned elsewhere (43) and according to PCR conditions in our previous study (44).

### 2.5 Quantitative real-time PCR for fungi

To detect the presence and quantify the number of fungi in each sample, specific primers were designed from identical regions of the internal transcribed spacer (ITS) of selected yeasts (Table 1). Real-time PCR was carried out in a 15 μL total volume by a Rotor-Gene Q (QIAGEN, Germany) real-time instrument with the following conditions: 7.5 μL of 2X real-time PCR Master Mix (BioFACT™, Korea), 0.5 μL of each primer (5 ρM), 3.5 μL of distilled water, and 3 μL of template DNA. Targeted fragments were amplified by cycling program: 95°C for 10 min followed by 40 cycles: 95°C for 25 s, 59°C for 30 s, and 72°C for 20 s and ramping from 70°C to 95°C at 1°Cs^−1^. An appropriate positive control and sterile distilled water as negative control were tested in each run. The melting profiles were also analyzed using Rotor-Gene Q software to exclude non-specific amplifications and primer dimers. In addition, a cycle of threshold (Ct) value of more than 35 or no amplification curve was considered negative.

**Table 1:** Primers used in this study.

Yeasts	Target region	Sequence	Fragment lengths
*C. albicans*	ITS	F: 5’-AACATTGCTTGCGGCGGTAA-3’ R: 5’-TACAACTCGGACGCCAAAGAC-3’	193 bp
*C. krusei*	ITS	F: 5’-TACTACACTGCGTGAGCGGA-3’ R: 5’-CTTTACACGTCGTCCGCTCC-3’	299 bp
*C. glabrata*	ITS	F: 5’-TTTGGTAGTGAGTGATACTCTCGT-3’ R: 5’-ACACTCACTTATCCCTCCCTAGA-3’	180 bp
*C. tropicalis*	ITS	F: 5’-TTGAACAAATTTCTTTGGTGGC-3’ R: 5’-GTCGCTTAAAATAAGTTTCCACG-3’	346 bp
*C. parapsilosis*	ITS	F: 5’-CCTTCTATATGGGGCCTGCC-3’ R: 5’-TGGAAGAAGTTTTGGAGTTTGTACC-3’	354 bp
*G. candidum*	ITS	F: 5’-AGTGAGGCTTCCGGATTGATTA-3’ R: 5’-CTTCCGCAGGTTCACCTACG-3’	126 bp
*S. cerevisiae*	ITS	F: 5’-GAGTTGTTTGGGAATGCAGCT-3’ R: 5’-ACCGAGGCAAGCTACATTCC-3’	299 bp
*C. neoformans*	ITS	F: 5’-GAGTTGTTTGGGAATGCAGCT-3’ R: 5’-ACCGAGGCAAGCTACATTCC-3’	265 bp
*Rhodotorula* spp.	ITS	F: 5’-TTAACTTGGAGCCCGAACTCTC-3’ R: 5’-CGAGAGCCAAGAGATCCGTT-3’	158 bp

Absolute quantification real-time PCR was used to quantify the number of fungi in each sample. Positive controls were selected from the collection bank of Tehran Medical Mycology Laboratory (TMML) located in the Dept. of Parasitology and Mycology, Tehran University of Medical Sciences, with collection numbers: TMML-1227, TMML-1226, TMML-1223, TMML-1218, TMML-1322, TMML-621, TMML-695, TMML-2651, and TMML-181 for *C. albicans, C. tropicalis, C. glabrata, C. parapsilosis, C. krusei, G. candidum, Rhodotorula* spp., *Cryptococcus neoformans,* and *S. cerevisiae*, respectively. For this purpose, 1 × 10^6^ were counted as follows: A loop full of a colony of each yeast was suspended in sterile PBS (pH = 7.5), and the number of yeasts was counted using spectrophotometry with a wavelength of 600. An optical density (OD) between 0.95 and 1.05 was considered 1 × 10^6^. Afterward, DNA was extracted from each yeast, and six serial dilutions with log ^−10^ were prepared from extracted DNAs.

Serial dilutions for each yeast were included in each real-time PCR experiment together with samples. A standard curve was drawn using serial dilutions to quantify the number of yeasts in each run. An R higher than 0.985 was considered as perfect.

### 2.6 Statistical analysis

A statistical analysis using the Chi-square test was conducted to compare the presence or absence of various fungi. The number of different fungi was compared using either the Mann–Whitney *U*-test or Kruskal–Wallis test. A logistic regression model was utilized to identify significant predictors for the existence of fungal species. In addition, a Spearman correlation coefficient was calculated to determine relationships. The R software, version 4.3.3, was used for all statistical analyses, and a *p*-value of less than 0.05 was considered statistically significant.

## 3 Results

**Figure 2:**
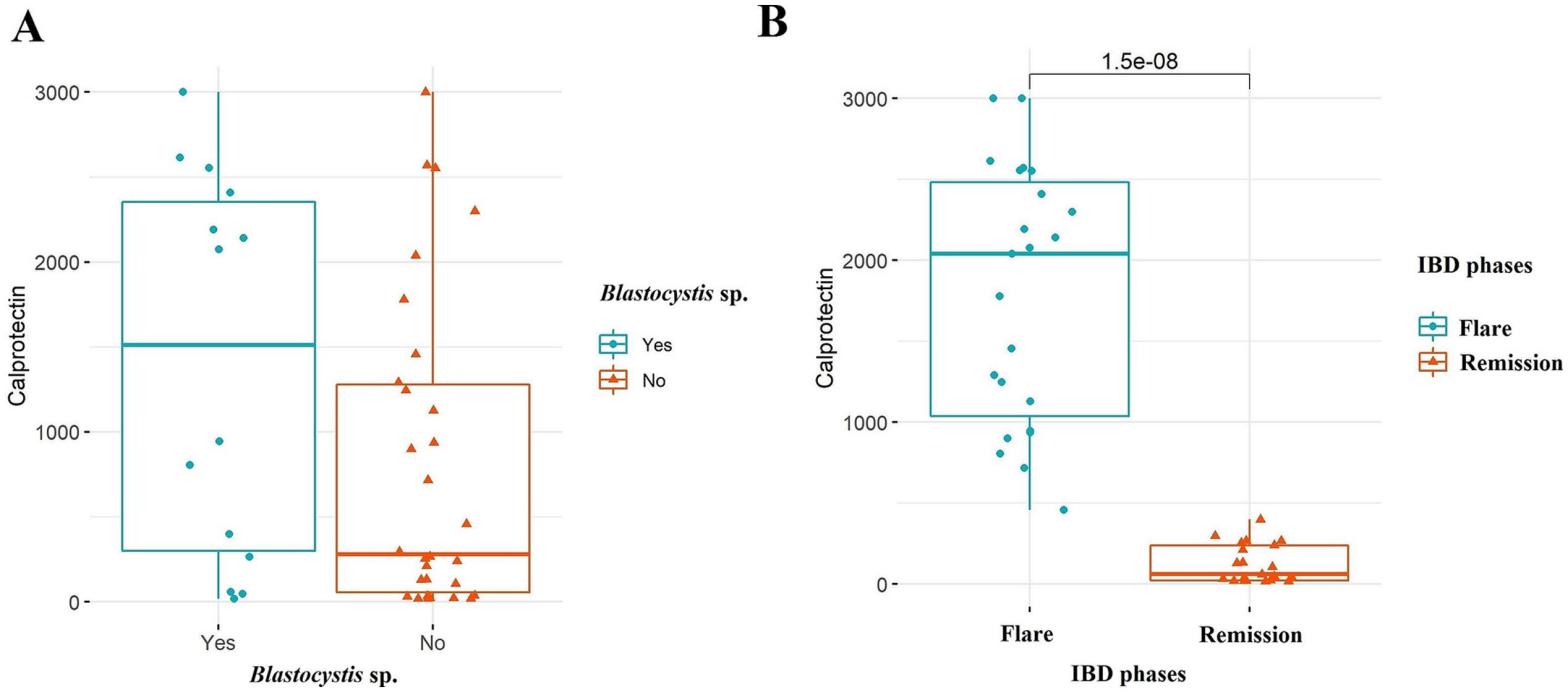
Comparison of fecal calprotectin levels among the IBD patients in **(A)** *Blastocystis* sp. and **(B)** IBD phases. Mann–Whitney *U*-test was used to analyze probable associations.

In total, 44 IBD patients, 20 healthy controls, and 27 IBS patients were included in our study, of which 45 and 46 were males and females, respectively, with an age range from 15 to 72 years. The mean age ± standard deviation (SD) of participants was 39.44 ± 9.91, 38.34 ± 13.2, and 43.00 ± 15.60 for IBS, IBD, and healthy subjects, respectively. According to the patient reports and observations, 17 and 10 IBS patients were classified as IBS-U (IBS-unclassified) and IBS-D (IBS-diarrhea), respectively. From 44 IBD patients, 39 (88.63%) and 5 (11.37%) suffered from UC and CD, respectively. Among IBD cases, 23 (52.27%) and 21 (47.73%) subjects were recorded as flare and remission phases, respectively (Table 2). According to the IBD activity scores extracted from the calprotectin values, 20, 7, 5, and 10 for patients were classified into 4 groups with calprotectin values >299, 300–999, 1,000–1999, and >2000 μg/g, respectively. The flare phase was determined based on the calprotectin scores recommended by the clinical consultant. The stool appeared formed and watery samples in 35 (79.55%) and 9 (20.45%) patients, respectively. All watery stool samples were seen in patients suffering from flare IBD. The presence of *Blastocystis* was identified among 15 (55.55%), 10 (50%), and 14 (31.82%) for IBS patients, healthy controls, and IBD patients, respectively. Accordingly, the mean ± SD of calprotectin in patients with flare and remission phases was 1787.24 ± 788.56 and 124.8 ± 119.07, respectively. As a fact, in IBD, there was a significant difference in the levels of fecal calprotectin between IBD patients in the flare phase and those in remission (*p-*value <0.001) (Figure 2). Interestingly, there was also no significant difference in fecal calprotectin levels in IBD patients with and without *Blastocystis* (*p-*value = 0.099) (Figure 2). The association between *Blastocystis* and activity scores released from fecal calprotectin values was also assessed, which was near-significant (*p-*value = 0.051). There was a significant association between the stool appearance and the presence of *Blastocystis* (*p-*value = 0.019).

**Table 2:** Demographic data of participated subjects.

Variables	IBS Patients (*n* = 27)	Healthy subjects (*n* = 20)	IBD patients (*n* = 44)
Gender			
Female	14	10	22
Male	13	10	22
Age			
Mean ± SD	39.44 ± 9.92	43.00 ± 15.60	38.34 ± 13.20
Min	20	15	57
Max	57	69	72
IBS subtypes			
IBS-D	10	–	–
IBS-U	17	–	–
IBD types			
UC	–	–	39
CD	–	–	5
IBD phases	–	–	–
Flare	–	–	24
Remission	–	–	20

IBS, irritable bowel syndrome; IBD, inflammatory Bowel diseases; IBS-D, IBS-diarrhea; IBS-U, IBS-unclassified; UC, ulcerative colitis; CD, Crohn’s disease.

### 3.1 Yeast distribution

Real-time PCR was used to identify yeasts in samples. Our results showed the presence of *C. albicans*, *C. glabrata, C. krusei, C. parapsilosis, S. cerevisiae, G. candidum,* and *Rhodotorula* spp. in samples. Accordingly, *S. cerevisiae* was the most prevalent yeast in IBS, IBD, and healthy subjects: 19/27 (70.37%), 25/44 (56.82%), and 17/20 (85%), respectively. In IBS patients, the detected yeasts were *C. albicans* (13/27; 48.14%)*, G. candidum* (12/27; 44.44%)*, C. glabrata* (3/27; 11.11%), and *Rhodotorula* spp. (1/27; 3.7%). *Saccharomyces cerevisiae* was the dominant yeast in IBD (25/44; 56.82%), followed by *C. albicans* (19/44; 43.18%), *G. candidum* (11/44; 25%), *Rhodotorula* spp. (7/44; 15.91%), *C. glabrata* (2/44; 4.54%), and *C. parapsilosis* (1/44; 2.27%). In healthy controls, *S. cerevisiae* (17/20; 85%), *C. albicans* (9/20; 45%), *G. candidum* (6/20; 30%), *C. glabrata* (2/20; 10%), and *C. krusei* (2/20; 10%) were detected. *Candida krusei* in IBD and IBS patients and *Rhodotorula* spp. and *C. parapsilosis* in healthy subjects were not detected. *Cryptococcus neoformans* and *C. tropicalis* were identified in neither IBD nor IBS and healthy subjects. In addition, 12, 4, and 3 of IBD, IBS, and healthy subjects were negative for all tested yeasts, respectively (Table 3). There was no significant association between the activity scores and the presence of each yeast. In addition, except for *C. glabrata* (*p-*value = 0.038), the association between the presence of yeasts and stool forms was not statistically significant. The probable association between fecal calprotectin score and the presence of yeasts was also investigated which was not statistically significant.

**Table 3:** Prevalence of tested yeasts in studied groups.

Study groups		*C. albicans*	*C. glabrata*	*C. krusei*	*Rhodotorula* spp.	*G. candidum*	*S. cerevisiae*
IBS	*Blastocystis* positive	8/15 (53.33%)	2/15 (13.3%)	0	1/15 (6.66%)	4/15 (26.66%)	11/15 (73.33%)
	*Blastocystis* negative	5/12 (41.66%)	1/12 (8.33%)	0	0	7/12 (58.33%)	8/12 (66.66%)
IBD	*Blastocystis* positive	8/14 (57.14%)	2/14 (14.30%)	0	4/14 (28.60%)	4/14 (28.60%)	9/14 (64.30)
	*Blastocystis* negative	11/30 (36.66)	0	0	4/30 (13.33%)	7/30 (23.33%)	16/30 (53.33%)
Healthy	*Blastocystis* positive	5/10 (50%)	2/10 (20%)	0	0	0	8/10 (80%)
	*Blastocystis* negative	4/10 (40%)	0	2/10 (20%)	0	6/10 (60%)	9/10 (90%)
Total		41	7	2	9	28	61

The Chi-square test was responsible for analysis.

Based on the presence of *Blastocystis*, *S. cerevisiae* (11/15; 73.33%)*, C. albicans* (8/15; 53.33%)*, G. candidum* (4/15; 26.66%)*, C. glabrata* (2/15; 13.33%), and *Rhodotorula* spp. (1/15; 6.66%) were detected in IBS patients. Except for *G. candidum* (7/12; 58.33%), the prevalence of all tested yeasts in *Blastocystis*-negative IBS patients was lower than in *Blastocystis*-positive IBS patients. A significant association between the prevalence of yeasts and the presence of *Blastocystis* was not seen in IBS patients (Table 3).

In IBD patients, the presence of *S. cerevisiae, C. albicans, G. candidum, Rhodotorula*, and *C. glabrata* in *Blastocystis*-positive samples was (9/14; 64.28%), (8/14; 57.14%), (4/14; 28.57%), (4/14; 28.57%), and (2/14; 14.28%), respectively. In IBD group without *Blastocystis*, the presence of *S. cerevisiae, C. albicans, G. candidum,* and *Rhodotorula* was confirmed in (16/30; 53.33%), (11/30; 36.66%), (8/30; 26.66%), and (4/30; 13.33%) patients, respectively (Table 3).

In healthy subjects, the prevalence of *S. cerevisiae*, *C. albicans*, and *C. glabrata* in *Blastocystis*-positive samples was (8/10; 80%), (5/10; 50%), and (2/10; 20%), respectively, while in *Blastocystis*-negative samples, *S. cerevisiae, C. albicans, G. candidum,* and *C. krusei* were detected in (9/10; 90%), (4/10; 40%), (6/10; 60%), and (2/10; 20%), respectively. In addition, the Chi-square test showed that the correlation between the prevalence of yeasts and the presence of *Blastocystis* was not significant in healthy subjects (Table 3). The presence/absence of *C. albicans* and *G. candidum* was not significantly different between patients with IBD, IBS, and the control groups. However, there was no significant difference in the presence of *S. cerevisiae* among patients (*p*-value = 0.077) (Table 4).

**Table 4:** Comparison of fungal distribution between IBD patients, IBS patients, and healthy controls using the chi-square test.

Categories	IBD	IBS	Control	*P*-value
*C. albicans*				
Yes	19 (43.2)	13 (48.1)	9 (45)	0.920
No	25 (56.8)	14 (51.9)	15 (55)	
*G. candidum*				
Yes	11 (25)	12 (44.4)	6 (30)	0.228
No	33 (75)	15 (55.6)	14 (70)	
*S. cerevisiae*				
Yes	25 (56.8)	19 (70.4)	17 (85)	0.077
No	19 (43.2)	8 (29.6)	3 (15)	

A logistic regression analysis was conducted to determine the presence of yeasts among patients (Tables 5, 6). The results revealed that IBD patients had a higher chance of having *S. cerevisiae* compared to the control group (OR (95% CI) = 4.15 (1.05, 16.37); *p-*value =0.042). The presence of *Blastocystis* also showed a near-significant relationship with *G. candidum* (OR (95% CI) = 2.69 (0.99, 7.30); *p-*value =0.053).

**Table 5:** Logistic regression analysis of the association between fungi and *Blastocystis* in patients.

Yeasts	Independent variables	Logistic Regression		
		OR	95% CI	*P-value*
*C. albicans*	Disorder (IBD vs. Control)	0.96	(0.32, 2.85)	0.944
	Disorder (IBS vs. Control)	0.91	(0.28, 2.94)	0.874
	*Blastocystis* (Yes/No)	0.54	(0.23, 1.28)	0.160
*G. candidum*	Disorder (IBD vs. Control)	1.55	(0.46, 5.21)	0.483
	Disorder (IBS vs. Control)	0.49	(0.14, 1.72)	0.265
	*Blastocystis* (Yes/No)	2.69	(0.99, 7.30)	0.053
*S. cerevisiae*	Disorder (IBD vs. Control)	4.15	(1.05, 16.37)	0.042
	Disorder (IBS vs. Control)	2.42	(0.55, 10.66)	0.242
	*Blastocystis* (Yes/No)	0.80	(0.31, 2.04)	0.636

**Table 6:** Logistic regression analysis of the association between fungi species among IBD patients with different phases and *Blastocystis*.

Yeasts	Independent variables	Logistic Regression		
		OR	95% CI	*P-value*
*C. albicans*	IBD phase (Flare/Remission)	0.50	(0.15, 1.75)	0.280
	*Blastocystis* (Yes/No)	0.48	(0.13, 1.79)	0.275
*G. candidum*	IBD phase (Flare/Remission)	0.41	(0.13, 2.29)	0.413
	*Blastocystis* (Yes/No)	0.82	(0.19, 3.63)	0.816
*S. cerevisiae*	IBD phase (Flare/Remission)	1.63	(0.48, 5.58)	0.438
	*Blastocystis* (Yes/No)	0.58	(0.15, 2.21)	0.424

**Figure 3:**
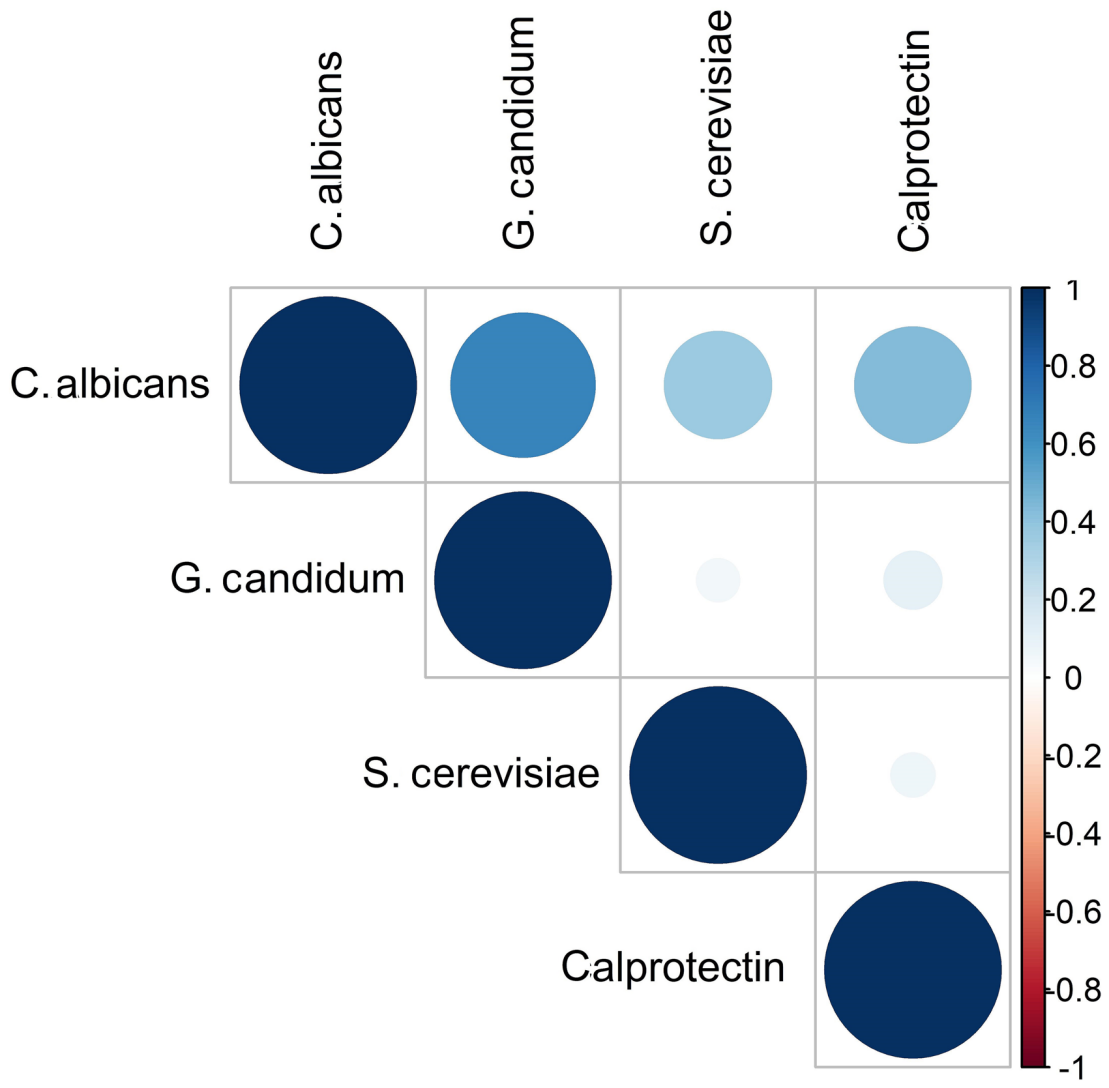
Correlation matrix diagram for the strength of the relationships between fungi species and fecal calprotectin among IBD patients. The size and the color intensity of the dark blue show an association between variables.

We did not find any significant associations between the presence of different yeasts and the IBD phases or *Blastocystis* among IBD patients. In addition, the coefficient correlation between *C. albicans* and calprotectin was near-significant (*r* = 0.433, *p-*value =0.064) (Figure 3).

### 3.2 Yeast quantification

**Figure 4:**
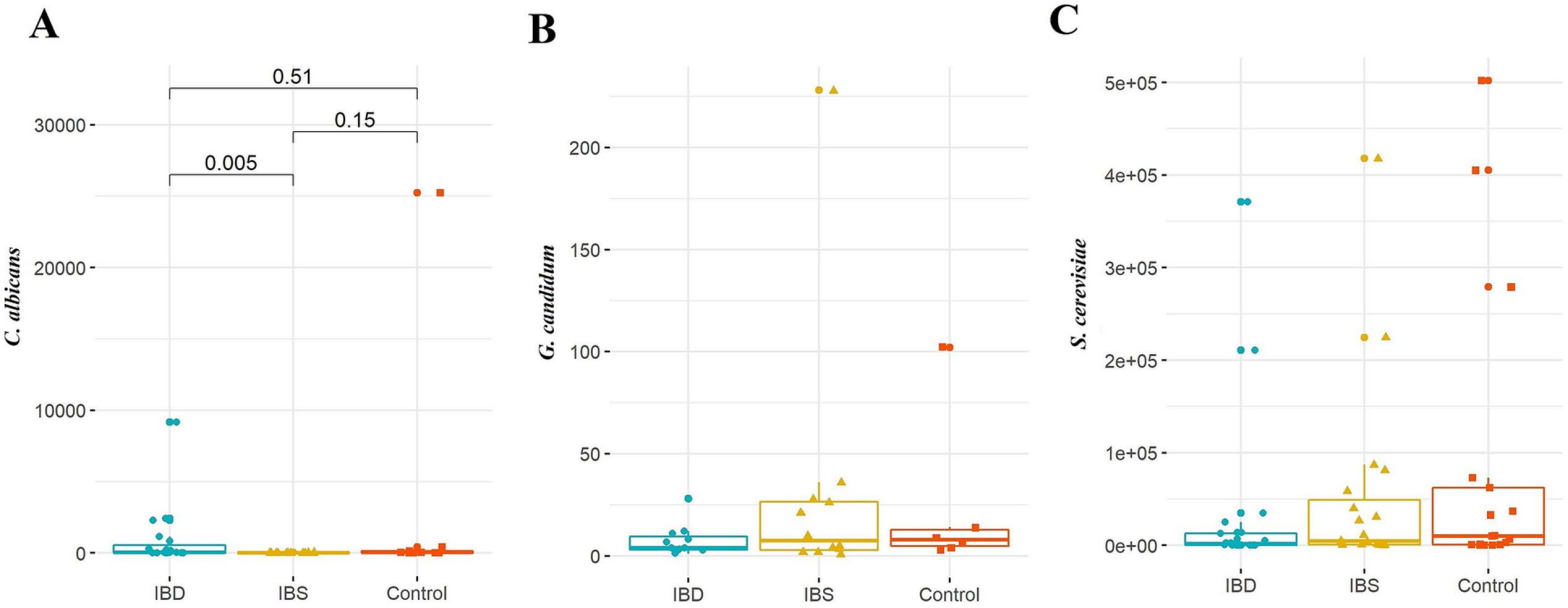
Comparison of fungal species: **(A)** *C. albicans*, **(B)** *G. candidum*, and **(C)** *S. cerevisiae* between IBD patients, IBS patients, and healthy controls. Kruskal–Wallis was used to evaluate the probable association between the quantity of selected yeasts and disorders. A *post-hoc* test was carried out to identify differences between groups.

When looking at the number of *C. albicans*, there was a significant difference among patients (*p-*value = 0.020), in which the source of difference was seen between IBD and IBS patients (*p-*value = 0.005). There were no statistically significant differences in the quantity of *G. candidum* and *S. cerevisiae* across the different disorders of the patients (Figure 4).

Regarding the presence of *Blastocystis*, a statistically significant association was only seen between the number of *C. albicans* and the sample groups. Accordingly, the number of *C. albicans* in IBS patients who carried *Blastocystis* was significantly lower than two other groups (*p-*value = 0.013) (Table 7).

**Table 7:** Median (min to max) of the quantity of tested yeasts based on the absolute quantitative real-time PCR.

Yeasts		IBS Median (min to max)	IBD Median (min to max)	Healthy Median (min to max)
*C. albicans*	*Blastocystis* positive	5.5 (1–55)	254 (5–9,156)	41 (6–25,230)
	*Blastocystis* negative	5 (1–29)	13 (2–2,425)	4 (1–421)
*C. glabrata*	*Blastocystis* positive	2 (2–2)	11095.5 (61–22,130)	827 (2–1,652)
	*Blastocystis* negative	1 (1–1)	–	–
*C. krusei*	*Blastocystis* positive	–	–	–
	*Blastocystis* negative	–	–	4.5 (4–5)
*G. candidum*	*Blastocystis* positive	3 (2–36)	7.5 (3–28)	–
	*Blastocystis* negative	10 (1–228)	4 (1–12)	7 (3–102)
*Rhodotorula* spp.	*Blastocystis* positive	–	3 (1–6)	–
	*Blastocystis* negative	1 (1–1)	1 (1–2)	–
*S. cerevisiae*	*Blastocystis* positive	3,843 (1–224,525)	1,605 (31–370,979)	8,217 (281–278,968)
	*Blastocystis* negative	22,186 (470–417,883)	1567.5 (1–34,963)	32,600 (1–501,855)

## 4 Discussion

The gut microbiota is a complex population of bacteria, viruses, protozoa, and fungi, whose number is approximately 10 times higher than the number of cells of host origin (45). It was estimated that only 0.1% of the gut microbiota are of eukaryotic origin, including protozoa and fungi (46, 47).

The role of mycobiome in GI disorders has significantly been highlighted in recent years. It has been suggested that although fungi and protozoa cover few portions of the gut microbiota, they are suggested to significantly shape the gut microbiota (10). A bacteria–fungi interaction was documented in GI disorders such as IBS (23) and IBD (16). It was demonstrated that gut microbiota dysbiosis may alter the fungal composition of the gut; however, this inter-kingdom correlation has been controversially depicted. For example, Hong et al. (23) proposed more susceptibility of gut mycobiome core compared to the gut bacteriome core in IBS-D patients, in which gut mycobiome changes could be considered a diagnostic signature. This finding confirmed the determinative role of gut mycobiome in presenting symptoms in IBS-like rats that suffered from visceral sensitivity (35). Such a co-variation between mycobiome and bacteriome was reported by Das et al. (36), who reported a co-variation between the gut mycobiome and bacteriome in IBS patients, which was suggested to be a potential clinical diagnostic value.

In IBS patients, *Blastocystis* is one of the most prevalent detected protozoa (27); therefore, investigating the interaction between this protist and prevalent yeasts in this group of patients could be interesting. In the current study, we failed to significantly correlate the prevalence of *S. cerevisiae* and *C. albicans*, two landmark yeasts of the gut, with the presence of *Blastocystis* in IBS patients, although our findings showed co-variation between these yeasts and *Blastocystis*, in which the prevalence of *S. cerevisiae* and *C. albicans* was increased from 41.6 and 66.66% in *Blastocystis*-negative to 53.3 and 73.3% in *Blastocystis*-positive subjects, respectively. In contrast, the co-variation between the presence of *Blastocystis* and the prevalence of *S. cerevisiae* and *C. albicans* was not observed in healthy subjects. Therefore, in addition to the available data indicating an increase in the presence of *S. cerevisiae* and *C. albicans* in IBS-like animal models (48), the gut condition and gut microbial disturbance in IBS subjects may increase eukaryotic–eukaryotic interactions. *Blastocystis* colonization seems to provide a favorable niche for the colonization of *S. cerevisiae* and *C. albicans* in IBS condition. However, the presence of *Blastocystis* increased and decreased the quantity of *C. albicans* and *S. cerevisiae*, respectively, regardless of the presence of IBS.

In IBD patients, a lack of co-variation was reported in CD patients between bacteria and fungi (16), while Imai et al. (49) showed mycobiome–bacteriome co-variation in CD patients compared to healthy and UC subjects. As a finding, although a statistically significant association between the presence of selected fungi and IBD was not seen, the quantity of *C. albicans* was correlated with the presence of IBD. In addition, the association between fecal calprotectin and the presence of *C. albicans* in IBD patients was close but not significant. Such a close association between *C. albicans* and IBD was documented by Yan et al. (50), who suggested a correlation between elevated levels of T helper 17 (Th17)-mediated immune responses and pathogenesis of IBD as well as the presence of *C. albicans.* Nevertheless, the lack of significant association between the presence of yeasts and the fecal calprotectin scores suggests that, most probably, there are more variables affecting the colonization of yeasts in the GI than just the increased number of Th17 cells. Interestingly, similar to *C. albicans*, the presence of *Blastocystis* in IBD patients was associated with elevated fecal calprotectin levels, despite it was insignificance.

On the other hand, *Blastocystis* has been associated with gut microbiota variation in healthy subjects and patients who suffer from GI disorders (40, 51–53). Owing to this, there is no knowledge of intra-kingdom interaction between fungi and eukaryotic microorganisms such as *Blastocystis*. Due to available studies, mining the correlation of *Candida* overgrowth with IBS-like symptoms (36, 54) and the high prevalence of *Blastocystis* in IBS patients (38, 39), the eukaryote–eukaryote interaction and synergistic effects of *Blastocystis* on the growth of *C. albicans* and the presentation of IBS symptoms should be considered. Furthermore, the quantity of *S. cerevisiae*, experienced as a potential probiotic in IBS patients to ameliorate the symptoms (55, 56), was reduced in the *Blastocystis*-positive group. This effect of *Blastocystis* on the richness of *C. albicans* and *S. cerevisiae* may rebut the term “healthy indicator” for this protozoan (41, 57, 58) not only in IBS patients but also in healthy subjects and IBD patients, in which the presence of *Blastocystis* increased and decreased the richness of *C. albicans* and *S. cerevisiae,* respectively, in healthy and IBD subjects.

This study is one of the first investigations evaluating the probable association between yeasts and *Blastocystis* in two significant GI disorders, IBS and IBD. Although this study is a pilot investigation, released results can provide interesting clue about intra-kingdom associations for further studies. However, the critical limitation of this study is the lack of a comprehensive molecular method for investigating a broad range of microorganisms. In addition, working on a higher number of samples can provide more accurate results. Another weakness of this study may be attributed to the lack of endoscopic scores that could help to extract more accurate data about the associations between clinical variables and yeast distribution in IBD patients.

## 5 Conclusion

In this study, regardless of the presence of *Blastocystis,* only the quantity of *C. albicans* was significantly different between IBS and IBD patients. Our findings showed a co-variation between the amount of *C. albicans* and the presence of *Blastocystis*. In contrast, the association between the number of *S. cerevisiae* and the presence of *Blastocystis* was reversed in all groups. The presence of *Blastocystis* was significantly associated with the stool forms, as well. Studies of mycobiome composition and intra-kingdom interaction between fungi and protozoa are limited. Although eukaryotes comprise a small portion of the gut microbiome, they are supposed to play a significant role in the development and symptoms of GI disorders. This investigation is a preliminary study mining eukaryote–eukaryote correlation in IBS and IBD patients. However, considering the probable determinative role of eukaryotes in shaping the gut microbiota composition, investigations of bilateral correlation between potential indicator microorganisms, such as *Blastocystis*, *C. albicans,* and *S. cerevisiae,* may provide interesting data.

## Data Availability

The original contributions presented in the study are included in the article/supplementary material, further inquiries can be directed to the corresponding authors.
